# Case report: Laparoscopic totally extraperitoneal repair of an obturator hernia with self-gripping mesh under spinal anaesthesia

**DOI:** 10.1016/j.ijscr.2019.07.048

**Published:** 2019-07-22

**Authors:** Wilson Petrushnko, Anna Isaacs, Tony Hackland, Michael Ghusn

**Affiliations:** a Department of Surgery, Upper Gastrointestinal Department, The Tweed Hospital, Australia; b Department of Anesthesia, John Flynn Hospital, Australia

**Keywords:** Case report, Obturator hernia, TEP, Self gripping mesh

## Abstract

**Introduction:**

Obturator hernias account for less than 0.073% of all hernias and less than 1.6% of all cases of mechanical bowel obstructions.

**Presentation of Case:**

We present a case of a 79 year-old elderly female with two recurrent bowel obstructions that have resolved with conservative management. On the third presentation we performed a totally extraperitoneal repair (TEP) with conscious sedation and a L3/4 spinal block. An obturator defect was patched with a self gripping mesh (progrip). The patient was discharged day 2 post operatively.

**Discussion:**

Laparoscopic surgery can be safely performed in high risk patients with careful monitoring. Laparoscopic surgery is usually associated with a shorter post-operative length of stay.

**Conclusion:**

This case demonstrates the successful but unconventional repair of an obturator hernia in a patient who had a high risk of significant morbidity and mortality with a more conventional anaesthesia and surgery.

## Highlights

• Laparoscopic repair of obturator hernia with self-gripping mesh can adopted to reduce the morbidity of open repairs.
• It is safe to perform laparoscopic TEP repairs under spinal anesthesia.

## 1 Introduction

Obturator hernias account for less than 0.073% of all hernias and less than 1.6% of all cases of mechanical bowel obstructions [1,2]. Obturator hernias have the highest rate of mortality amongst all abdominal wall hernias.

They can be a diagnostic dilemma, as unlike other hernias in the inguinal regions, there is often no palpable mass. Female patients have a six fold increase in developing an obturator hernia compared to males [3]. A contributing factor is the thin body habitus and they are often seen in the elderly. The following case report has been reported in line with the SCARE criteria [4].

## 2 Presentation of case

**Fig. 1:**
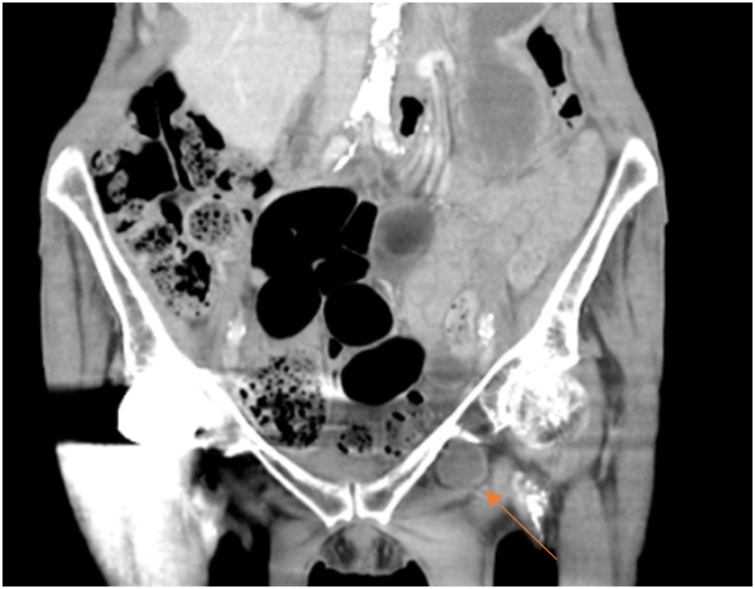
CT scan demonstration of a left obturator hernia.

We present a case of a 79 year-old elderly female. She had previously had two bowel obstructions in a short period that resolved with conservative management. She presented a third time and was referred to the authors. A CT scan was performed which revealed signs of a small bowel obstruction due to small bowel in a left sided obturator hernia (Fig. 1).

The decision to operate was carefully balanced with the patient’s co-morbidities. The patient was ASA 4E, weighed 29 kg, with severe COPD requiring continuous home oxygen. The operative strategies were carefully discussed with the anaesthetist and we decided to proceed with a laparoscopic totally extraperitoneal (TEP) repair. The patient had conscious sedation with a titrated propofol infusion & L3/4 spinal block with 1.5 ml of plain 0.5% Bupivacaine aiming for a T6 level block. However, sensory level was at T11, so the initial infra-umbilical port was too painful & a modified lower camera port around the suprapubic area (T12) was used successfully (Fig. 5). Insufflation pressure of 7 mmHg was used and no reverse Trendelenburg adopted in an attempt to minimise cardiovascular (CV) and cardiorespiratory compromise. A further 5 mm working port 2 cm inferior to the camera port and a second 5 mm working port medial to the left ASIS was adopted. The port sites were infiltrated with bupivicaine/adrenaline 0.2%.

**Fig. 2:**
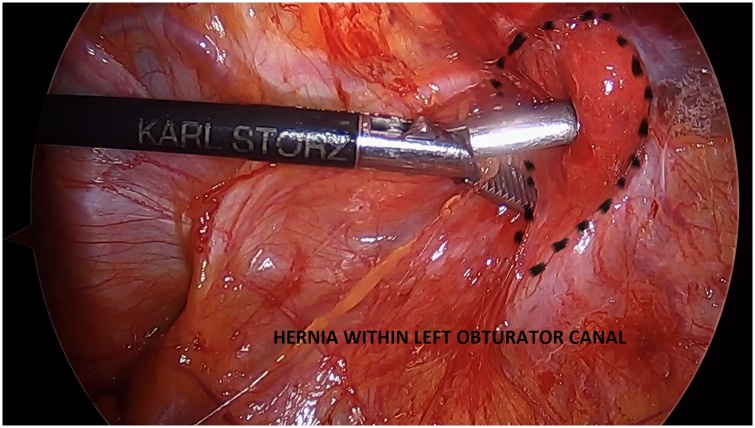
Reduction of small bowel within the left obturator canal.

**Fig. 3:**
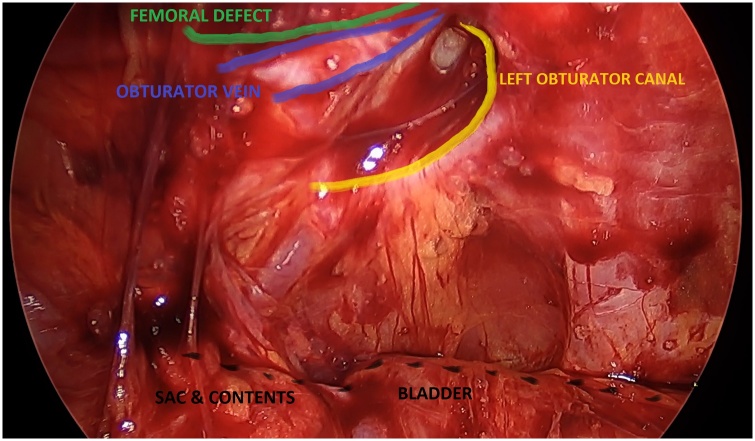
Left sided obturator defect.

**Fig. 4:**
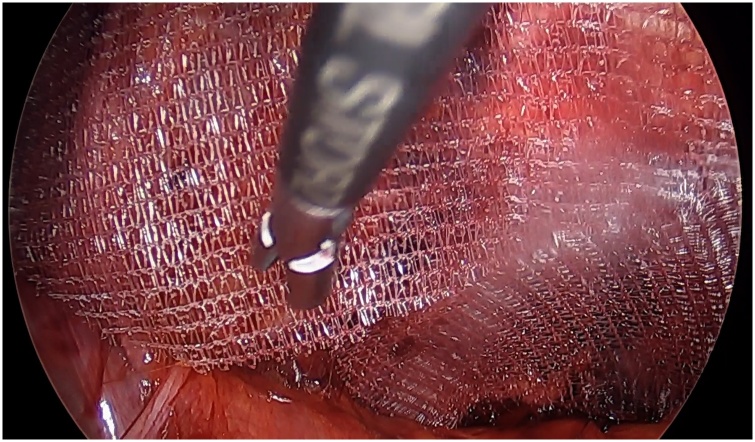
Demonstrating the self-gripping mesh repair of the left sided obturator hernia.

Under vision, we found an obturator hernial defect containing small bowel within a peritoneal sac (Fig. 2). The hernia was reduced and there was no evidence of bowel was ischemia (Fig. 3). The hernia was repaired with two pieces of self gripping mesh (Progrip) sized 4 × 6 cm (Fig. 4).

**Fig. 5:**
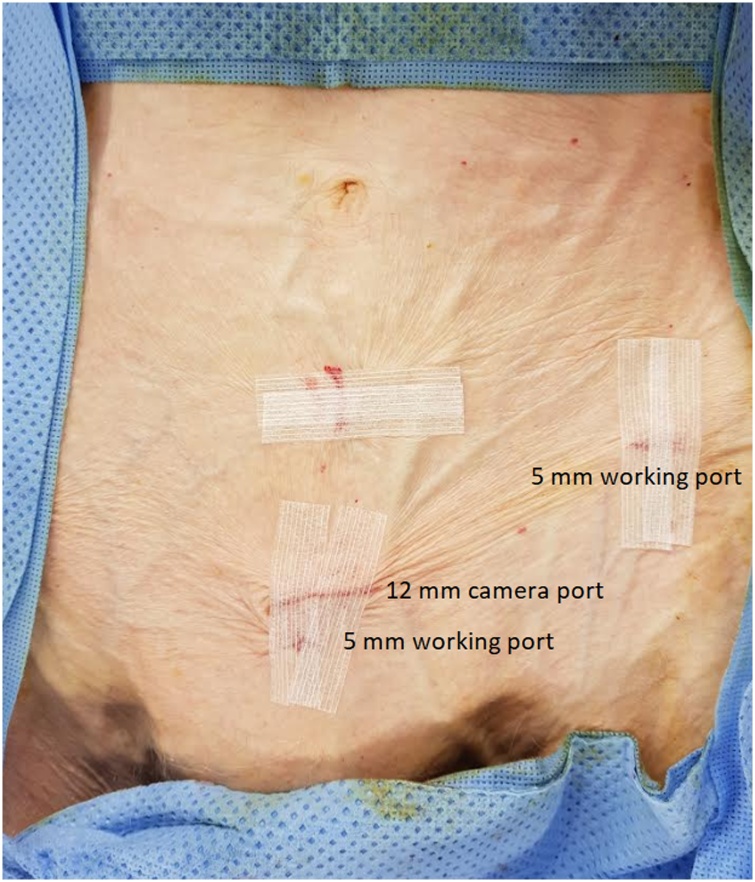
Port placement: Suprapubic camera port placement.

The Patient was observed in a high dependency unit for 24 h and was discharged home day 2 post-operatively. Only oral paracetamol was used for analgesia post operatively.

## 3 Discussion

Obturator hernias were first described in 1724 by Arnaud de Ronsil [5]. They are rare, representing 0.073% of all hernias repaired [1]. Females are more at risk due to a broader pelvis, a larger obturator canal, and a tendency for the pelvic peritoneum to become lax [3,6].

A high index of suspicion is required, particularly in elderly women with recurrent small bowel obstructions. A delay in accurate diagnosis can result in significant morbidity and mortality [6]. The use of CT scans can be helpful in establishing a diagnosis of an obturator hernia [7,8]. In our case the patient had two small bowels obstructions in a short period of time and a CT was helpful in diagnosing the obturator hernia. A definitive repair of the obturator hernia was required in this case.

Obturator hernia repair has always been difficult and technically challenging with several different approaches, but laparoscopy has the advantage of improved vision in the pelvis compared to open approaches [9]. Laparoscopic surgery can be safely performed in high risk patients with careful monitoring [10]. Laparoscopic surgery is usually associated with a shorter post-operative length of stay [11]. Laparoscopic TEP repair under spinal anaesthesia has been described previously [[12], [13], [14]]. A randomized control study evaluated the surgical outcome of laparoscopic TEP inguinal hernia repair under spinal anaesthesia (SA) versus repair under general anaesthesia (GA) and found no difference in complications but an improved pain score in the group who underwent SA [15].

The advantages of using a self gripping mesh were demonstrated in our case. Self gripping meshes have been found advantageous in reducing the incidence of chronic pain post inguinal hernia repairs [16] without significant long term complications [17].

Our experience with self gripping mesh has been that it is very useful in repairing hernias bordering bone and vascular structures (i.e. sub-xiphoid, morgagni, femoral, obturator and suprapubic). The use of self-gripping mesh often avoids the need for further fixation in these hernias thus decreasing pain and risk of injury.

## 4 Conclusion

This case demonstrates the successful but unconventional repair of an obturator hernia in a patient who had a high risk of significant morbidity and mortality with a more conventional anaesthesia and surgery. Surgeon experience with laparoscopic TEP hernia repair and use of self-gripping mesh allowed a tailored surgical approach in a high-risk patient.

## Sources of funding

No funding was obtained for the research.

## Ethical approval

Ethics approval was not required for the publication of the case report. Written consent was obtained from the patient.

## Consent

Written consent was obtained from the patient to publish the case report and all images associated.

## Author contribution

The following manuscript titled “Laparoscopic totally extraperitoneal repair of an obturator hernia with self-gripping mesh under spinal anaesthesia.’ has been equally prepared and edited by all the listed authors: Wilson Petrushnko, Anna Isaacs, Tony Hackland and Michael Ghusn.

## Registration of research studies

The case report was not registered.

## Guarantor

Dr W. Petrushnko.

Dr M. Ghusn.

## Provenance and peer review

Not commissioned, externally peer-reviewed.

## Declaration of Competing Interest

Dr Ghusn – undertakes paid consultancy work for Medtronic.
